# Cultural trauma as a fundamental cause of health disparities

**DOI:** 10.1016/j.socscimed.2021.114574

**Published:** 2021-11-17

**Authors:** Andrew M. Subica, Bruce G. Link

**Affiliations:** a University of California, Riverside School of Medicine, Department of Social Medicine, Population, and Public Health, 900 University Ave, Riverside, CA, 92521, USA; b University of California, Riverside School of Public Policy, 900 University Ave, Riverside, CA, 92521, USA

**Keywords:** Health disparities, Cultural trauma, Historical trauma, Social determinants

## Abstract

Health disparities disproportionately affect minority cultural groups (e.g., Indigenous, immigrant, refugee) worldwide; enduring across time, disease states, and risk factors despite co-occurring advancements in health and medicine. Fundamental cause theory holds that important social factors (e.g., socioeconomic status, stigma, racism) produce health disparities by restricting equitable access to health-protective resources. Yet, extant literature has not utilized fundamental cause theory to describe the health disparities impact of cultural trauma: an overwhelming, often ongoing physical or psychological assault by an oppressive dominant group on another group’s cultural resources through force, threats of force, or oppressive policies. This paper presents a novel conceptual model detailing cultural trauma and the mechanisms through which it may disrupt health and create disparities by damaging three health-protective cultural resources: cultural modes, institutions, and lands. Following cultural trauma, we propose affected groups are socially disadvantaged and exposed to pervasive stress, stigma, and diminished resources, perpetuating health disparities across generations. Consequently, cultural trauma may represent an unrecognized fundamental cause of health disparities, offering potential avenues for promoting health equity through targeted research, interventions, and policies.

## 1. Introduction

Many minority cultural groups worldwide—including Indigenous, immigrant, refugee, and sexual minority populations—experience profound cultural traumas (e.g., colonization, genocide, hate crimes). Health disparities consist of inequities in health resulting from social disadvantage (Adler, 2009), and are caused by powerful social factors such as socioeconomic status, stigma, and racism that disadvantage and stress minority group individuals (Hatzenbuehler et al., 2013; Link and Phelan, 1995; Williams and Collins, 2016)—increasing their overall risk for illness and death despite stark changes in diseases (e.g., cholera, HIV/AIDs), risk factors (e.g., unsanitary living conditions, poor diet), and medical treatments over time (Link and Phelan, 1995). In this paper, we propose that because cultural trauma may also generate social disadvantage, stress, and mental and physical health problems in minority cultural populations, it may reflect an underrecognized driver of health disparities.

## 2. Collective trauma and culture

The purpose of this paper is to complement the existing literatures on collective, historical, intergenerational, and racial trauma—which have largely focused attention on these traumas’ physical, psychological, or existential harms—by presenting a novel theoretical model linking cultural trauma to health disparities from a resource deprivation/loss and social disadvantage perspective; which has not been posited previously. To do so, we situate these collective traumas in fundamental cause theory (Link and Phelan, 1995); an important piece of canon in the social determinants of health literature (Clouston and Link, 2021). Consequently, this paper does not focus on the devastating physical (e. g., torture, death) or psychosocial (e.g., psychological injury) impacts of collective trauma (which are already well described), but will illuminate how these collective traumas may influence health disparities by disadvantaging cultural groups through the damaging/suppression of health-protective resources.

Conceptually, “culture” is a broadly defined construct labeled by Markus and Kitayama “an untidy and expansive set of material and symbolic concepts … that give form and direction to behavior” (Markus and Kitayama, 2010, p.422), with culture generally considered the shared beliefs, attitudes, norms, practices, institutions, and policies of a particular nation, people, or other social group (Betancourt and López, 1993; Markus and Hamedani, 2007). Thus, “cultural groups” refer to diverse social groups defined by shared characteristics including race/ethnicity, religion, nationality, or sexual orientation. Far from being static, cultures are dynamic entities that respond reflexively to changes in environment, resources, and threats; adapting fluidly to afford their members a shared source of resilience, identity, meaning, and connection. Cultures are also resilient, changing in response to migration, acculturation, globalization, and acts of oppression and violence from other groups.

Drawing from this conceptualization of culture as well as current definitions of collective and historical trauma, we define cultural trauma *as an overwhelming and often ongoing physical or psychological assault or stressor perpetuated by an oppressive dominant group on the culture of a group of people sharing a specific shared identity/affiliation* (e.g., *race/ethnicity, nationality, religion)* (Evans-Campbell, 2008; Kohn and Reddy, 2006; Stamm et al., 2004). In the literature, current definitions of collective and historical trauma largely focus on the psychological or existential responses to a mass traumatic event with collective trauma being defined as the psychological reactions to a traumatic event shared by a group of individuals that becomes ingrained in the group’s collective memories (Hirschberger, 2018). Similarly, historical trauma has been conceptualized as a multigenerational trauma inflicted on a group of people with a shared identity/affiliation that encompasses their psychological and social responses to the traumatic event (Evans-Campbell, 2008). Many collective and historical traumas inflicted upon specific groups (e.g., Indigenous, refugee) may meet our cultural trauma definition and criteria. If this occurs, cultural trauma is considered to encompass these collective and historical traumas. However, we differentiate cultural trauma from prior definitions/conceptualizations of collective and historical traumas by centering the focus and impact of the traumatic event on a group’s *culture/cultural resources* vs. their psychological or physical well-being; as in collective and historical trauma (Evans-Campbell, 2008; Brave Heart and DeBruyn, 1998; Brave Heart, 1999; Hirschberger, 2018).

To differentiate *cultural* trauma from *interpersonal* trauma, whereas interpersonal trauma involves an assault by a person on an individual’s health, cultural trauma involves an assault by a dominant group on an individual’s culture—through force, threats of force, or oppressive policies—for the purposes of damaging, devaluing, or destroying that culture to advance the dominant group’s interests in gaining key resources (e.g., natural, labor) or status/reputation (e.g., colonial empires). Cultural trauma may overlap with interpersonal trauma, as in culturally motivated physical violence (e.g., genocide, hate crimes) toward members of a minority cultural group. But cultural trauma diverges from interpersonal trauma by accounting for how culturally motivated physical violence *and* non-physical assaults/stressors (e.g., racial discrimination, internment), driven by animus toward a group’s culture vs. a specific individual, may generate lasting health disparities that impact future generations not exposed to the original cultural violence/assault.

We introduce this new cultural trauma conceptualization for two reasons. First, current clinical definitions of trauma do not match the reported trauma experiences of many minority groups (Andermahr, 2015; Evans-Campbell, 2008; Krieg, 2009), which include ongoing persecution, discrimination, and retraumatization (Craps, 2013; Krieg, 2009). Second, the empirical evidence indicating that cultural trauma impacts health (Hartmann and Gone, 2014; Krieg, 2009; Whitbeck et al., 2004) is distributed across different constructs (e.g., collective vs. historical vs. intergenerational trauma), each describing separate aspects of the trauma experience that are often specific to different cultural groups (Sotero, 2006). We contend this lack of a unifying definition or model has arguably concealed cultural trauma’s true influence as a fundamental cause of health disparities.

## 3. Fundamental causes

According to fundamental cause theory, health disparities persist due to underlying social factors that disadvantage certain groups in accessing resources for protecting health and avoiding disease (Gee and Ford, 2011; Link and Phelan, 1995; Williams et al., 2019). As these resources can be used flexibly to avoid risks for, or reduce the impact of, multiple diseases across multiple situations, Phelan et al. (2010) labeled them ‘flexible resources’.

To comprise a fundamental cause, a social factor must satisfy three criteria (Link and Phelan, 1995; Phelan et al., 2010). First, it must impact multiple health outcomes in a population through multiple risk factors. Consequently, to be considered a fundamental cause, cultural trauma must be linked to numerous health disparities as in the case of American Indians, whose traumatic displacement and ensuing cultural loss instigated multiple health disparities that persist to this day (e.g., heart disease, suicidality, alcohol use disorders) (Brave Heart et al., 2011; Gone et al., 2019; Subica and Wu, 2018).

Second, the proposed factor must embody access to flexible resources (e.g., knowledge, money, power, prestige, beneficial social connections, freedom) (Phelan et al., 2010; Phelan and Link, 2015). For instance, during the past century, cultural traumas in the form of discriminatory U.S. housing practices (e.g., redlining, mortgage foreclosure, blockbusting) served to maintain health disparities and economic deprivation in African American communities by barring individuals from accruing critical health-protective resources such as property and intergenerational wealth (Jackson, 1980; Mehlhorn, 1998; Saegert et al., 2011; Satter, 2009).

Third, the proposed factor’s production of health disparities must be reinforced over time through multiple replaceable mechanisms (Phelan and Link, 2015). Continuing with our example, after explicit forms of cultural trauma toward African Americans (e.g., slavery, Jim Crow laws, lynching) were outlawed through policy changes, other forms of cultural trauma such as mass incarceration, housing discrimination, and police violence that “*transmit the legacy of the oppressive past of slavery and Jim Crow*” (Feagin, 2000, p. 145) were instituted to perpetuate African Americans’ unequal access to cultural and other flexible resources (Saegert et al., 2011; Satter, 2009). Additionally, because health disparities generated by fundamental causes are rooted in inequities in flexible resources, health disparities cannot be eliminated by intervening on a specific disease or risk factor (Phelan et al., 2010).

To date, several fundamental causes—e.g., socioeconomic status (SES; Link and Phelan, 1995), stigma (Hatzenbuehler et al., 2013), racism/racial segregation (Phelan and Link, 2015; Williams and Collins, 2016)—have been codified in the literature. Herein, we present evidence and a theoretical model implicating cultural trauma as an unrecognized fundamental cause of health disparities; harming health by assaulting a group’s essential cultural resources.

## 4. Cultural resources

**Fig. 1.**
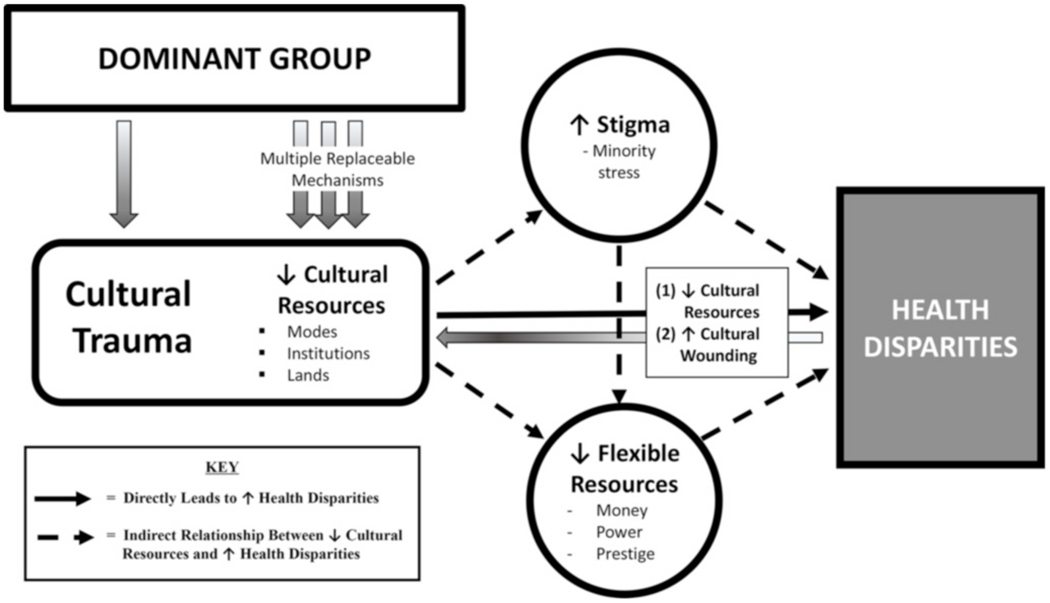
Cultural trauma conceptual model.

Central to our cultural trauma model (Fig. 1) is the empirically grounded notion that culture represents an unrecognized flexible resource for health that is foundational to human survival. Accordingly, we identified from the literature three cultural resources that when disrupted via cultural trauma may trigger an intergenerational cascade of negative health outcomes. Like water to a fish, these cultural resources permeate human behavior and the social environment so deeply that they are often taken for granted. This paper closes this gap by merging existing research into a new model that recognizes three essential cultural resources for health: *cultured modes, institutions*, and *lands*.

### 4.1. Modes

Kitayama et al. (2007) define ‘cultural modes of being’ as a group’s languages, norms, customs, values, and artifacts that construct both the internal and social worlds of group members. Modes are essential for healthy functioning as they organize and pattern one’s thoughts, feelings, and actions, define one’s place in the world relative to others (e.g., self-esteem, ethnic identity, cultural worldview), and frame one’s social interactions to meet their basic, social, and health needs (Kitayama et al., 2007; Markus and Kitayama, 2010; Ryba et al., 2016). In essence, cultural modes encompass our ways of living, behaving, and experiencing the world to navigate society and fulfill our needs.

When cultural trauma damages a group’s cultural modes, psychological studies have shown that healthy functioning can be disrupted as modes serve crucial psychological functions including protecting against stress and anxiety, promoting self-regulation, and facilitating effective adaptation/response to external stressors (Markus and Kitayama, 2010; Norris et al., 2002; Salzman, 2001; Savickas, 2005). For example, empirical studies of Terror Management Theory (Greenberg et al., 1986) strongly support the functioning of cultural modes as an important protective factor against anxiety (especially related to vulnerability and death), with the invalidation or loss of cultural modes having potentially devastating effects on individuals’ self-esteem and anxiety (Greenberg et al., 1990, 1997; Pyszczynski et al., 2004). In addition, according to Evans-Campbell’s (2008) historical trauma framework, the devaluing/destruction of cultural modes by a dominant group may profoundly shift cultural roles and identities, leading some survivors to experience elevated suicide, depression, substance use, chronic grief, and PTSD susceptibility (Brave Heart et al., 2011; Cook et al., 2003; Duran et al., 1998).

For instance, the Western colonization of Hawai’i wrought sweeping changes to Hawaiian social and cultural order through oppressive policies that destroyed native cultural modes including the banning of the Hawaiian language, prohibiting traditional spiritual ceremonies and rituals, and mixing sacred male and female work and living roles (Bushnell, 1993; Cook et al., 2003). According to Cook et al. (2003), this trauma to Hawaiian cultural modes generated cultural confusion and spiritual damage, which manifests as health disparities among present-day Native Hawaiians (Braun et al., 1995; Look and Braun, 1995; Mau et al., 2009).

### 4.2. Institutions

Institutions are the second cultural resource damaged by cultural trauma, and refer to the sociocultural systems—and policies upholding these systems—that establish social order in a society and govern people’s behaviors and expectations (North, 1990). These systems cover all areas of social and community life and include family, economic, legal, educational, religious, political, and health systems (Miller, 2003; North, 1990). According to social status research, when individuals are afforded positive status within their cultural institutions, institutions protect against stress and support health (Demakakos et al., 2008; Hatzenbuehler et al., 2013; Sapolsky, 2004). But when institutions relegate individuals into lower statuses, institutions generate stress and derail health (Adler, 2009; Sapolsky, 2004). For example, Sapolsky’s review of social status and health showed that forced subordination by dominant individuals within social hierarchies inhibits subordinates’ health by imposing physical and psychological stressors *and* resource inequities on subordinates; which may be perpetuated through unequal institutionalized policies/actions including denial of flexible resources (Link and Phelan, 1995) and racism (Bailey et al., 2017; Williams et al., 2019).

Additionally, when a dominant group damages a group’s cultural institutions via cultural trauma, survivors may be brought into the dominant group’s institutions in subservient roles and status (Berry, 1989); exposing these individuals to potential acculturative stress, social disadvantage, and health-depriving discrimination and racism (Berry, 1989; Williams and Berry, 1991). For instance, after requiring Aboriginal groups to endure forced relocation, many Western nations consigned surviving children to residential schools to be “civilized” into Western institutions (e.g., family, religious, health systems); deepening poverty, unemployment, and ensuing health disparities such as tuberculosis, substance use, obesity, and early mortality in Aboriginal communities (Haskell and Randall, 2009; MacDonald and Steenbeek, 2015; Phillips-Beck et al., 2019; Walls and Whitbeck, 2012).

Additionally, research suggests systematic disadvantaging within cultural institutions may foster health disparities by fomenting racism; worsening health through multiple mechanisms including racial trauma (Comas-Díaz et al., 2019), social deprivation (Bailey et al., 2017), residential segregation (Williams and Collins, 2016), and unequal health care access (Flores, 2010; Smedley et al., 2003). For example, following extensive cultural trauma and disadvantaging in U.S. social, economic, educational, and criminal justice institutions, African Americans currently experience heavy disparities in morbidity and mortality (Cunningham et al., 2017; Huie et al., 2003; Orsi et al., 2010; Read et al., 2005; Williams et al., 2019).

### 4.3. Lands

Cultural lands is the third trauma-impacted resource, and refers to the material resources (e.g., physical property, housing, healthy foods, transportation, wealth) necessary to sustain health in a given society. As noted by scholars of colonialism and historical trauma, when a cultural group’s lands gain a dominant group’s interest, the dominant group may wield cultural trauma to dispossess the cultural group of its lands (Duran et al., 1998; Brave Heart and DeBruyn, 1998; Howe, 2002; Kohn and Reddy, 2006); potentially draining survivors of their health while restricting future opportunities to restore their cultural lands—and health—to pre-trauma levels (Evans-Campbell, 2008; Saegert et al., 2011).

Within the literature, this cultural trauma appears to occur both through physical dislocation from native lands via force, genocide, or disease (Evans-Campbell, 2008; Krieg, 2009), or discriminatory policies that strip groups of existing cultural lands (i.e., material resources) while blocking access to future lands and other flexible resources (Fullilove, 2001; Williams and Collins, 2016). For forcibly dislocated groups (e.g., Indigenous populations, refugees/asylum seekers), this traumatic displacement presents a massive stressor that impacts mental and physical health in the short-term and long-term (Bogie et al., 2015; Brave Heart and DeBruyn, 1998; Thomas and Thomas, 2004). For instance, American Indians’ traumatic relocation from economically valuable lands to barren reservations (1) ruptured their cultural ways of life; (2) created a deep psychological injury and sense of traumatic loss; and (3) robbed them of health-protective flexible resources (Kirmayer et al., 2014; Walls and Whitbeck, 2012)—instigating a cultural genocide (Brave Heart et al., 2011) that underlies the glaring health disparities afflicting present-day American Indian communities (Jones, 2006; O’Connell et al., 2010; Subica et al., 2017).

Similarly, social psychiatrist Mindy Fullilove (2001) noted that the 1949 U.S. Urban Renewal Act-related dispossession of the lands of many flourishing Black communities intensified existing health disparities by (1) generating prolonged stress, grief, and trauma-related symptoms in displaced residents (Fried, 1963); (2) segregating residents into areas of concentrated poverty with poor access to flexible resources (Williams and Collins, 2016); and (3) forcing residents to expend major financial capital to resettle, casting subsequent generations into economic disadvantage (Fullilove, 2001).

## 5. Multiple replaceable mechanisms

In fundamental cause theory, the mechanisms enacted by a dominant group to suppress access to flexible resources must be replaceable over time (Link and Phelan, 1995; Phelan and Link, 2015). More pointedly, when new opportunities to reinforce the effect of a fundamental cause on health disparities materialize, new mechanisms will reliably emerge to support or strengthen the relationship (Phelan and Link, 2015).

In cultural trauma, due to culture’s dynamic nature and cultural groups’ resilience in responding to cultural threats and oppression, we theorize dominant groups must continually implement ‘multiple replaceable mechanisms’ to maintain their advantage in flexible resources by quelling minority groups’ efforts to reclaim their cultural resources and subsequent health (Fig. 1). As stated by Phelan et al. (2008), dominant groups achieve this by keeping other groups down (to exploit or dominate them), in (by enforcing dominant group cultural modes), or away (physically or socially distancing from cultural groups perceived as health threats).

There are multiple ways that dominant groups may prevent culturally traumatized groups from restoring or gaining needed cultural and other flexible resources. It can happen at the structural level through policies such as establishing American Indian boarding schools and reservations, or police surveillance of minority communities. It can happen at the interpersonal level via direct actions such as verbal threats, hate crimes, police violence, or discrimination in employment and housing. It can also be expressed by doing nothing when harm is experienced such as when low-income minority children suffer malnutrition and poor health due to food insecurity (Thomas et al., 2019) or American Indian women go missing or harmed while receiving minimal mainstream outcry or attention (Lucchesi and Echo-Hawk, 2018). What each mechanism achieves is a reinvigorated hierarchy in which the dominant group replaces older or failing mechanisms with new ones that maintain their ability to limit other groups from exerting their will and restoring their cultural and other flexible resources; keeping them down, in, or away. For example, following the repeal of policies that limited equal distribution of economic and social privileges to Asian Americans such as the Chinese Exclusion Act, anti-miscegenation laws, and the internment of Japanese Americans (Higginbotham and Kopytoff, 1988; Nagata, 1998; Soennichsen, 2011; Sohoni, 2007), new mechanisms of anti-Asian suppression and violence predictably emerged (Chang, 1998); gaining increased virulence and frequency during the COVID-19 era (Stop AAPI Hate, 2021).

## 6. Cultural trauma mechanisms

Fig. 1 displays three empirically supported mechanisms through which our model proposes cultural trauma influences health disparities. The first mechanism operates directly by damaging health-protective cultural resources and generating psychologically and physically harmful cultural wounding while the other mechanisms operate indirectly by stigmatizing traumatized groups with status loss and stereotyping, and denial/depletion of flexible resources, respectively. As our indirect mechanisms’ influence on health disparities have been previously detailed in earlier papers by Hatzenbuehler et al. (2013) and Phelan et al. (2010), this paper will elucidate cultural trauma’s role in exacerbating these mechanisms.

### 6.1. Direct mechanism: cultural resource loss and cultural wounding

By damaging a group’s cultural resources (modes, institutions, lands), our model holds that cultural trauma denies members essential flexible resources that support and protect against stress, mental illness, and disease. As social scientists and Indigenous scholars have observed, damage to one’s culture directly harms health by denying individuals vital culture-related health protective factors including (1) cultural/ethnic identity and pride (Crocker et al., 1994; Phinney and Ong, 2007); (2) cultural coping and healing strategies (Gone, 2013); and (3) supportive social, community, and family networks (Krieg, 2009; Norris et al., 2002).

Building on existing research, cultural trauma may also directly harm health by creating deep, intergenerational ‘cultural wounding’ (Evans-Campbell, 2008; Brave Heart and DeBruyn, 1998). Labeled a “soul wound” by Duran et al. (1998), this wounding has been characterized as a deep psychological injury that directly compromises people’s sense of well-being, safety, self-regard, and coping. It is physically and emotionally destructive, manifesting in problems such as depression, anxiety, shared posttraumatic stress, and substance use (Brave Heart et al., 2011; Gone and Alcántara, 2007; Whitbeck et al., 2004) that lead to adverse health outcomes (e.g., diabetes, suicide, cancer) (Bezo and Maggi, 2018; Goodkind et al., 2012; Shrira et al., 2011). These wounds may be felt collectively across generations, impacting offspring through exposure to daily reminders of the cultural trauma including discrimination and loss of traditional languages, family systems, and spiritual/healing practices (Evans-Campbell, 2008; Subica and Wu, 2018; Whitbeck et al., 2004).

### 6.2. Indirect mechanisms: stigma and flexible resources

Our model further proposes that cultural trauma may affect health disparities via two indirect mechanisms (1) stigmatization of affected groups; and (2) decreased access to flexible resources.

Stigma is the labeling, stereotyping, separation, status loss, and discrimination of disadvantaged groups by those in power to achieve their desired goals (Link and Phelan, 2001). In our model (Fig. 1), we assert that dominant groups wield stigma to maintain dominance over minority groups’ cultural and other flexible resources. According to Link and Phelan’s (2001) theories on stigma, the dominant group may exercise stigma using multiple mechanisms such as anti-immigrant media and messaging (Morey, 2018) and resource-reducing discrimination in employment, housing, and education (Hatzenbuehler et al., 2013) to marginalize minority groups with status loss and negative labels/stereotypes (Link and Phelan, 2001). A vivid example is the descriptive labeling/othering of certain minority groups in Western countries as “immigrants,” “refugees,” “queer,” or “terrorists” vs. their appropriate cultural affiliations (e.g., Latin American, sexual minority, Muslim).

From the psychological literature, we further posit dominant groups are motivated to wield stigma to justify their cultural trauma by propagating narratives that denigrate, blame, or cast as inferior affected groups—mimicking perpetrators’ use of “victim-blaming” attributions to justify traumatizing behaviors (Harsey et al., 2017; Henning et al., 2005). Noteworthy dominant group justifications for inflicting cultural trauma in the literature include discriminatory narratives such as “We’re civilizing the savages” by European colonists (Wallace, 2010), “We’re the superior race” by the Third Reich (Tenenbaum, 1956), and “They don’t belong here” against immigrant populations (Morey, 2018). Following stigma exposure, individuals may experience minority stress—a stigma-related chronic, additive stress (on top of general life stressors) associated with reduced mental and physical health (Hatzenbuehler et al., 2013; Meyer, 2003) in stigmatized cultural groups such as sexual minorities (Hatzenbuehler, 2009; Operario et al., 2015).

In our model’s second indirect mechanism, cultural trauma may perpetuate health disparities by decreasing affected groups’ access to flexible resources (e.g., money, power, prestige) (Link and Phelan, 1995; Phelan and Link, 2015) through (1) cultural resource loss; and (2) stigma-related minority stress and disadvantaging. For example, the culturally traumatic denial of marriage rights (a valued cultural institution) to same sex couples denied millions of sexual minorities access to important marriage-related flexible resources (e.g., employer-sponsored health insurance, partner-inherited wealth) (Encarnación, 2014; Gonzales and Blewett, 2014; Perone, 2015).

## 7. Intergenerational cultural trauma

The intergenerational transmission of cultural trauma has been clearly observed in multiple populations including Holocaust survivors (Yehuda et al., 2008), refugees (Field et al., 2013; Sangalang and Vang, 2017), combat veterans (Dekel and Goldblatt, 2008), and Indigenous populations (Brave Heart, 2003; Kirmayer et al., 2014). Extensive research has highlighted several mechanisms through which intergenerational trauma transmission may affect health disparities including impaired parenting and parental modeling of maladaptive post-traumatic behavioral patterns (Auerhahn and Laub, 1998; Brave Heart, 2003; Krieg, 2009), secondary traumatization through transmission of trauma narratives to offspring (Brave Heart et al., 2011; Whitbeck et al., 2004), and potential epigenetic changes that increase susceptibility to mental and physical disorders/diseases (e.g., depression, metabolic syndrome, cardiovascular disease)(Bierer et al., 2014; Darity et al., 2001; Heckman and Payner, 1989; Yehuda et al., 2016).

Supported by this evidence, our model proposes that health disparities caused by cultural trauma are intergenerationally transmitted through the passing down of (1) cultural wounding and loss; (2) social disadvantage; and (3) biological vulnerabilities to disorders/diseases. These risk factors then increase members’ exposure to persistent stress, stigma, racism, and diseases, which accumulate over time to perpetuate health disparities (Gone et al., 2019; Sotero, 2006).

## 8. Interventions

Several intervention approaches may play a role in addressing health disparities by targeting cultural trauma through direct intervention or policy change. The first seeks to restore damaged cultural modes via cultural/legacy interventions (Brave Heart et al., 2011; Gone and Alcántara, 2007; University of Calgary, 2012) that build cultural identity, pride, and knowledge, and facilitate cultural healing through racial socialization, traditional practices (e.g., diets, ceremonies), and cultural education (Anderson and Stevenson, 2019; Cook et al., 2003; Gone, 2013). An excellent example is Australia’s Yiriman Project where Aboriginal elders take at-risk youth on ‘back-to-country’ walking trips containing cultural activities (e.g., fishing, land management, story-telling) to reinvigorate lost Aboriginal culture, lore, and identity (Palmer et al., 2020). Additionally, resilience interventions engaging at-risk Black youth in Africentric practices and rites of passages have led to increased self-esteem, ethnic identity, and decreased high-risk behaviors (e.g., substance use, aggression/violence) (Belgrave et al., 2004; Harvey and Hill, 2004; Jackson et al., 2010) while *Aloha Āina* (caring for the land) interventions have strengthened cultural identity and self-esteem in Native Hawaiian youth (Mokuau, 2011).

A second approach employs community mobilization/capacity building interventions to restore groups’ cultural resources and protective factors (e.g., community networks, collective efficacy) (Sotero, 2006). One promising example is community organizing-based health promotion (Subica, Grills, Douglas, et al., 2016), which engages and mobilizes disadvantaged residents to rectify community inequities in flexible resources by building community power to advocate for health-promoting policy changes to the social and built environment (Subica, Grills, Villanueva, et al., 2016). Increasing community power via grassroots interventions may also counteract the social paralysis/political disengagement of many traumatized groups that may sustain inequities in flexible resources and health over time (Degagné, 2007; Fullilove, 2001).

Unfortunately, targeting cultural trauma using these therapeutic and structural approaches alone places the onus on the *oppressed* to “fix the problems” created by dominant groups’ traumatizing behaviors. Therefore, approaches should also intervene on *oppressors* through policy interventions that challenge the systems of domination/power that maintain health disparities through the suppression of cultural and other flexible resources. Examples include restoring disrupted cultural modes and institutions by establishing trusts and educational systems that uplift minority cultures/languages, as in Hawai’i’s Kamehameha Schools that was formed in 1887 to counteract the severe socioeconomic and educational disadvantages facing Native Hawaiians (Serrano et al., 2007). To promote access to cultural lands and other flexible resources, policy interventions may also take the form of equality generators such as baby bonds (Hamilton and Darity, 2010; Zewde, 2020), universal basic income (Haagh, 2019; Hoynes and Rothstein, 2019), or reparations for culturally traumatized populations including Jewish Holocaust survivors (Ludi, 2012), the Indigenous Sámi of Europe (Errico and Hocking, 2012), Japanese Americans (Howard-Hassmann, 2004), and Africans (Spitzer, 2002).

Finally, an important intervention approach involves direct public actions (e.g., protests) that raise the dominant group’s awareness of the harmful effects of ongoing traumas (e.g., racially-biased police violence) (Edwards et al., 2019; Nix et al., 2017; Williamson et al., 2018). This policy-focused approach is exemplified by the global Black Lives Matters movement (Jee-Lyn García and Sharif, 2015), which in disrupting existing law enforcement and political systems of domination has forced a reckoning and deeper understanding among many dominant group members of the entrenched systemic problems and oppressive policies (e.g., racism, police violence) (Political Polling U.S., 2020) that contribute to illness and death in many cultural populations.

## 9. Conclusion

Fundamental cause theory explains health disparities’ persistence over time despite major changes in risk factors and diseases (Phelan et al., 2010). Presented evidence indicates cultural trauma meets all fundamental cause criteria, profoundly shaping the intergenerational social and health trajectory of affected populations by (1) impacting multiple mental and physical health outcomes through multiple mechanisms (e.g., cultural wounding, stigma, biological vulnerabilities); (2) restricting access to health-protective cultural and other flexible resources; and (3) reproducing health disparities over time through multiple replaceable mechanisms.

At the same time, we do not consider cultural trauma to be the sole driver or root cause of health disparities, as cultural trauma often overlaps and interacts with other fundamental causes (e.g., racism, stigma, socioeconomic status) and social or environmental factors (e.g., inadequate healthcare access, poor air and water quality) in a complex, synergistic fashion to produce and maintain health disparities over time. Notably, there may be cases where cultural trauma overlaps with structural racism—i.e., race-based discrimination practiced by a dominant group within racialized social systems (Bonilla-Silva, 1997). However, two key points serve to differentiate cultural trauma from structural racism. First, while racism is inherently race-based, cultural trauma is culture-based and can therefore account for the occurrence of health disparities in cultural groups affiliated by shared identities other than race such as sexual orientation, gender identity, immigration status, religion, and physical and psychiatric disability. Second, for cultural trauma to reflect a fundamental cause, it must (a) involve a clearly identifiable physical or psychological assault/stressor; (b) be produced by a dominant group motivated to achieve its desired ends (e.g., material resources, status); and (c) directly impact health by damaging the affected group’s cultural modes, institutions, or lands. In contrast, racism (a) may be more insidious than cultural trauma and does not need to involve an overt assault/stressor (e.g., racial microaggressions); and (b) is caused by a dominant group primarily motivated by an ideology of inferiority, with the dominant group (c) exerting its power and control over resources by categorizing and ranking other people they perceive as inferior into social groups by “race” (Bonilla-Silva, 1997; Williams et al., 2019; Williams and Mohammed, 2013).

As a new model, limitations exist that should be addressed in future studies. First, because our goal was to introduce cultural trauma’s influence on health disparities from a fundamental cause perspective, we could not review many of the biological, psychosocial, and existential effects/processes reported in the literature on collective, historical, and racial traumas. Second, we presented our model as a generalized theory of cultural trauma in order to allow others to tailor it to the unique trauma experiences and outcomes of specific groups in ways that we could not. We encourage researchers to test, challenge, and improve our model so as to better measure and apprehend the full impact of cultural trauma on diverse groups. We particularly encourage individuals to consider ways to conceptualize and assess the deleterious effects of cultural trauma not only on larger cultural groups, but also subcultures within these groups that may be differentially affected by cultural traumas. Yet, despite these limitations, we believe our model may offer researchers new insights into why health disparities persist across time, place, and generations, and potentially inform future studies and interventions to reduce health disparities.

In closing, our paper suggests that cultural trauma may be an unrecognized fundamental cause of health disparities for the numerous cultural groups worldwide that have endured traumatic assaults on their cultural modes, institutions, or lands. Therefore, reducing health disparities may require a multipronged approach focused on restoring access to cultural/flexible resources while also healing these traumas’ intergenerational physical and psychological consequences. However, because oppressive mechanisms are replaceable, eradicating health disparities may require enacting policies designed to curtail dominant groups’ ability to exploit and control the resources of others to prevent cultural trauma and advance health equity for all groups.
